# To be or not to be a piRNA: genomic origin and processing of piRNAs

**DOI:** 10.1186/gb4154

**Published:** 2014-01-27

**Authors:** Adrien Le Thomas, Katalin Fejes Tóth, Alexei A Aravin

**Affiliations:** 1 California Institute of Technology, Division of Biology, 147-75, 1200E California Blvd, Pasadena, CA 91125, USA; 2 Université Pierre et Marie Curie, Ecole Doctorale Complexité du Vivant, 75005, Paris, France

## Abstract

Piwi-interacting RNAs (piRNAs) originate from genomic regions dubbed piRNA clusters. How cluster transcripts are selected for processing into piRNAs is not understood. We discuss evidence for the involvement of chromatin structure and maternally inherited piRNAs in determining their fate.

## Introduction

At the core of diverse RNA interference pathways operating in different species from Bacteria to Metazoa lies a ribonucleoprotein complex consisting of a small RNA, responsible for target recognition, and a member of the Argonaute protein family, carrying the effector function. In animals, three major classes of small RNA have been identified: the microRNA (miRNA), the small interfering RNA (siRNA) and the Piwi-interacting RNA (piRNA) [1]. miRNAs and siRNAs have important roles in post-transcriptional regulation of gene expression and defense against exogenous viral agents, respectively [2]. siRNAs and miRNAs are processed from double-stranded or hairpin precursors by the type III ribonuclease Dicer. The piRNA pathway silences transposable elements (TEs) in the gonads of Metazoa and acts at both the transcriptional and the post-transcriptional level [3-5]. Compared with siRNAs and miRNAs, the biogenesis of piRNAs is far less well elucidated. In mouse and *Drosophila*, piRNAs are derived from long precursor transcripts that originate from distinct genomic regions dubbed piRNA clusters [3]. What marks transcripts from these regions for processing into piRNAs is not fully understood. Here we summarize current knowledge about piRNA sources and biogenesis in mouse and *Drosophila*, and discuss possible mechanisms of how piRNA precursors are discriminated from other cellular transcripts.

## Genomic origin of piRNAs

miRNAs are encoded by specific genes that code for individual or just a few small RNA sequences, resulting in a total of a few hundred different miRNA species in both *Drosophila* and mouse. In contrast, piRNAs are very diverse: hundreds of thousands of unique piRNA sequences do not show any structure or sequence motif similarities, except for a bias for a uridine residue at the first base [3]. Mapping of these piRNA sequences to the genome revealed that piRNAs come from two types of genomic locations: the first and main source is discrete genomic loci, called piRNA clusters, whereas a smaller fraction of piRNAs map to a handful of protein-coding genes [6]. In *Drosophila*, piRNA clusters are strongly enriched in repetitive sequences, predominantly transposon remnants, and are devoid of protein-coding genes. They can span up to 200 kb and are located in pericentromeric and subtelomeric regions. Clusters are arranged in two constellations based on the orientation of their transcription: unidirectional clusters are transcribed in only one direction, whereas bidirectional clusters are transcribed convergently from two ends. Interestingly, the two cluster types differ in their expression pattern. Unidirectional clusters are expressed in the somatic follicular cells of the *Drosophila* ovary, whereas bidirectional clusters are transcribed in germline-derived nurse cells. P-element insertion at the beginning of the unidirectional *Flamenco* cluster disrupts piRNA expression up to 200 kb downstream of the insertion site, arguing for the presence of a single promoter responsible for the transcription of the whole cluster [3].

The second, far less abundant, source of piRNAs are protein-coding genes. Some piRNAs map to the 3′ untranslated region (UTR) of genes. The most prominent genic piRNAs in *Drosophila* come from the gene encoding the transcription factor Traffic Jam (*tj*) [7]. Interestingly, insertion of a heterologous sequence (encoding green fluorescent protein) into the 3′ UTR of *tj* generates abundant piRNAs from the inserted sequence, indicating that the whole transcript is recognized for processing [8].

In mouse, the piRNA population can be separated into two groups according to the time of their expression during spermatogenesis: pre-pachytene piRNAs resemble *Drosophila* piRNAs and silence TEs [9], whereas pachytene piRNAs, which start to be expressed at the pachytene stage of meiosis, are devoid of transposon sequences and their function is still unknown [9]. Pachytene piRNA clusters are either unidirectional or bidirectional; in the latter case, RNAs are transcribed from a central promoter in two opposite directions. It was recently shown [10] that transcription of both types of pachytene piRNA clusters is triggered by the binding of the transcription factor A-myb to a conserved sequence motif in the promoter. Combined analysis of high-throughput RNA-sequencing (RNA-Seq), cap analysis of gene expression sequencing (CAGE-Seq) and polyadenylation site sequencing (PAS-seq) data revealed that pachytene cluster precursors contain a 5′ cap and a 3′ poly(A) tail and are transcribed by RNA polymerase II, therefore resembling normal genic transcripts [10].

Based on informatic analysis, piRNA clusters in mouse and *Drosophila* do not show any primary sequence or secondary structure motifs that would clearly identify them as piRNA precursors, raising the question of how transcripts from piRNA clusters are recognized as precursors for piRNA processing.

## Processing of piRNAs

As piRNAs can originate from single-stranded RNA precursors, their processing differs from that of siRNAs and miRNAs. miRNA precursors contain hairpin structures, which are recognized and excised by the endonuclease Drosha. Subsequently the miRNAs are excised from the hairpins by Dicer, the same enzyme that processes long double-stranded RNAs into mature siRNAs. Dicer is a type III RNA endonuclease that is specific to double-stranded RNA and absolutely necessary for siRNA and miRNA production [11]. In contrast, processing of piRNAs is independent of Dicer [12].

**Figure 1:**
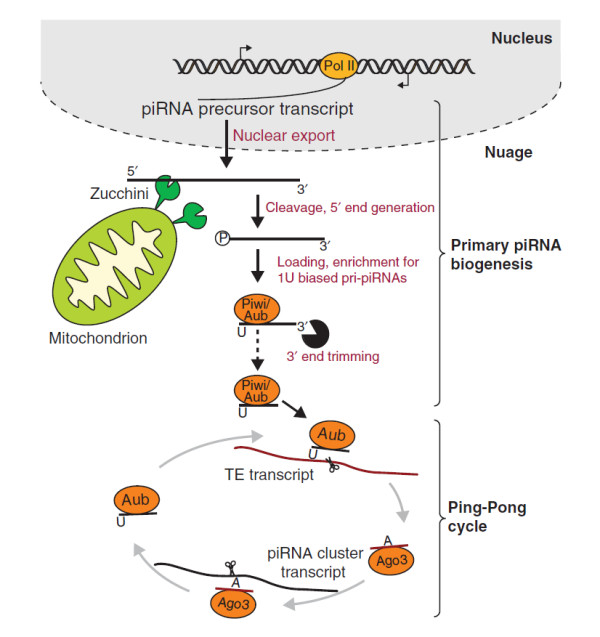
**piRNA biogenesis.** Two pathways, primary piRNA biogenesis and the Ping-Pong cycle, have been implicated in generation of piRNAs in *Drosophila* germ cells, whereas only primary piRNA biogenesis operates in follicular cells. Long RNAs transcribed from piRNA cluster regions are exported from the nucleus to nuage granules, where many protein components involved in the piRNA pathway localize and where piRNA biogenesis is believed to occur. During primary piRNA biogenesis long piRNA precursors are cleaved by an endonuclease, possibly Zucchini, located in the outer membrane of mitochondria, generating the 5′ end of the future piRNA. The cleaved transcript is loaded into Piwi proteins (Piwi and Aub) and then trimmed from the 3′ end by an unidentified trimmer nuclease to its final length. In the Ping-Pong cycle, Aub loaded with piRNAs recognizes and cleaves complementary RNAs such as transposon (TE) mRNAs or transcripts derived from the opposite strand of the same piRNA cluster. This cleavage produces the 5′ end of a new piRNA that is loaded into Ago3 and in turn can induce cleavage of complementary RNA. This generates a new piRNA that is identical in sequence to the piRNA that initiated the cycle.

The machinery involved in piRNA processing is largely unknown. It was proposed that long single-stranded transcripts are cleaved by an endonuclease to generate the 5′ end of the mature piRNAs [13]. Recently, a combination of genetic, biochemical and structural data identified Zucchini as a candidate nuclease for piRNA 5′ end processing [14,15] (Figure 1). Zucchini (the fly ortholog of the mouse MitoPLD) is a member of the phospholipase D family of phosphodiesterases and resides in the outer membrane of mitochondria. It cleaves the RNA leaving a 5′ phosphate residue characteristic for piRNAs. Following this cleavage, which generates the 5′ end of piRNAs, the piRNA precursors are thought to be loaded into Piwi proteins before their 3′ end is trimmed by a yet unidentified factor that was named trimmer [16]. Interestingly, each Piwi protein is associated with piRNAs of a distinct size range [3,17]. It was proposed that trimmer stops when it reaches the region of the RNA protected by the footprint of the Piwi protein, generating piRNAs of different lengths specific to each Piwi protein [16]. After piRNAs are trimmed to the final size, the 3′ end of the RNA is 2′–*O*-methyl-modified by Hen1, resulting in mature piRNAs [18]. piRNAs have a strong bias for a uridine residue as their 5′ nucleotide. Currently, the origin of this bias is unknown: it can be caused by preferential cleavage by Zucchini or due to selective incorporation of piRNAs containing a 5′ uridine into Piwi proteins. The latter model is supported by the observation that the 5′ binding pocket of many Argonaute proteins can sense the first base [19,20].

Many proteins that were implicated in piRNA biogenesis localize to nuage granules, cytoplasmic granular structures that are tightly associated with the outer nuclear membrane [21]. Accordingly, it is believed that many or all steps of piRNA biogenesis happen in nuage granules. In *Drosophila*, nuage granules contain two cytoplasmic Piwi proteins, Aubergine (Aub) and Argonaute3 (Ago3). Informatic analysis of piRNAs present in these two proteins revealed that Piwi proteins themselves can serve as nucleases that generate the 5′ end of new piRNAs, in a process that was named the Ping-Pong amplification cycle (Figure 1) [3,22]. Aub-loaded piRNAs recognize complementary transcripts (derived from active TEs or the opposite strand of the same piRNA cluster) and Aub cleaves them 10 nucleotides from the 5′ end of the original piRNA. This forms the 5′ end of a new piRNA, which is then incorporated into Ago3 and trimmed as described above. Ago3-associated piRNAs can in turn recognize complementary transcripts and cleave them, resulting in generation of new piRNAs that are identical in sequence to the initial piRNA that started the circle. Importantly, the Ping-Pong amplification loop is sensitive to target transposon expression and therefore might lead to amplification of piRNAs that target active elements.

It is essential that cells generate a proper pool of piRNAs that can target potentially dangerous transposons yet inhibit processing of transcripts from normal coding and non-coding genes. Next we discuss possible mechanisms that might be responsible for differentiation between sequences that are meant to be processed into piRNAs and other cellular transcripts.

## Selection of piRNA precursors

The fact that only a very specific subset of transcripts is processed into piRNAs indicates that some features of either the genomic locus or the transcript itself must differentiate piRNA precursors from other cellular RNAs. Three hypotheses can be proposed on how piRNA-producing loci are identified. First, distinct sequence or structure motifs in precursor transcripts or DNA inside or around clusters could signal the biogenesis machinery to process the transcript into piRNAs. Second, the chromatin structure of piRNA clusters could be marking these genomic regions for differential processing of their transcripts. Finally, piRNAs that are inherited from the previous generation could be responsible for selection of regions for piRNA processing in the progeny.

*Caenorhabditis elegans* piRNAs, called 21U-RNAs, are individually encoded by separate genes, all of which contain an octamer motif roughly 40 bp upstream of the piRNA sequence that is recognized by the Forkhead family of transcription factors [23]. Transcription by RNA polymerase II starts 2 bp upstream of the 5′ end of mature 21U-RNAs, generating capped piRNA precursors that are 26 nucleotides long [24]. Although the machinery involved in 21U-RNA processing is not known, the unique sequence motif required for their transcription might also signal their biogenesis.

In flies and mice, no unique sequence motifs have so far been identified in or around piRNA clusters. Although A-myb has binding motifs in the promoters of pachytene clusters in mouse, this motif is also present at promoters of several protein-coding genes whose transcripts are not processed into piRNAs, indicating that binding of A-myb cannot be a signal that discriminates piRNA loci. Similarly, no distinct structural motifs have been identified within piRNA clusters. Unlike in the case of siRNAs, there is no phasing of piRNAs: although they have a strong preference for uridine at their 5′ base position, piRNAs can start at any nucleotide within the cluster. Insertion of a heterologous sequence into a cluster leads to piRNA production from this sequence, indicating that any sequence can be processed into piRNAs [8]. These data argue against the existence of distinct sequence or structure motifs in piRNA loci that specify transcripts for piRNA processing in flies and mice, leaving the two alternative hypotheses as the more viable options.

### The role of chromatin structure in defining piRNA-producing loci

piRNA-producing loci could be specified by a unique chromatin structure. Chromatin structure has been mostly linked to transcriptional regulation of the underlying DNA. However, chromatin might also have an impact on the post-transcriptional fate of transcripts. How is this possible? Either by regulating processes that happen co-transcriptionally, such as splicing and polyadenylation [25], or by marking newly transcribed RNAs with specific proteins or modifications. For example, a recent study showed that in fission yeast transcription from heterochromatic loci (marked by the histone H3 lysine 9 trimethylation (H3K9me3) mark and the associated heterochromatin protein 1 (HP1)) leads to dissociation of HP1 from chromatin and its binding to nascent RNA. This induces the degradation of the heterochromatic transcript [26].

**Figure 2:**
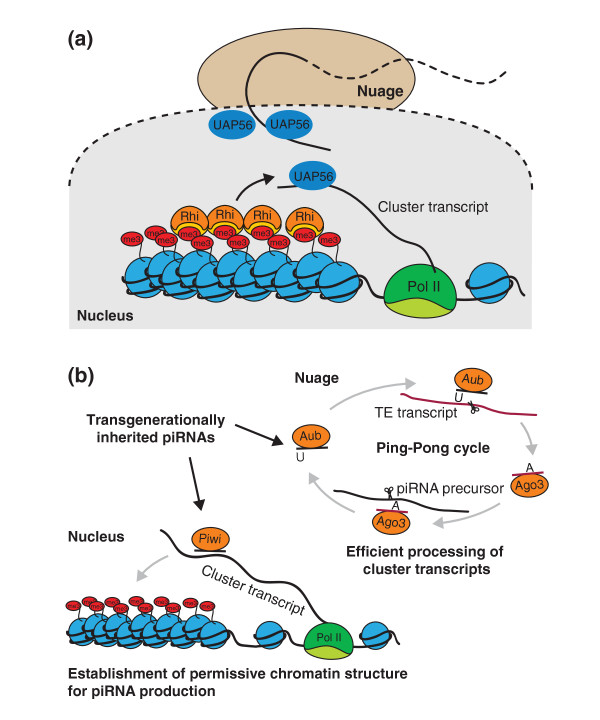
**Potential models for selecting transcripts for piRNA processing. (a)** A unique chromatin state of piRNA-producing loci can guide channeling of RNA transcribed from these regions to the piRNA processing machinery. Bidirectional piRNA clusters are enriched in the HP1 homolog Rhino. Rhino colocalizes with UAP56, which binds long piRNA precursors and shuttles them from the nucleus to cytoplasmic nuage granules. These interactions could facilitate targeted export of cluster transcripts directly to the processing machinery. **(b)***Trans*-generationally inherited piRNAs can guide selection of the transcripts processed to piRNA. Maternally transmitted piRNAs loaded into Aub and Ago3 complexes can initiate processing of complementary transcripts through the Ping-Pong cycle. Alternatively, inherited piRNAs bound to the nuclear Piwi protein could identify nascent transcripts and induce deposition of chromatin marks such as H3K9me3 that lead to processing of transcripts into piRNAs using the pathway shown in (a).

Increasing evidence indicates that piRNA clusters in *Drosophila* do have a unique chromatin environment. Most piRNA clusters are found at the boundary of euchromatic and heterochromatic regions in the pericentromeric and subtelomeric regions of the genome [3]. piRNA clusters show characteristics of heterochromatin, such as an enrichment of the repressive H3K9me3 mark and HP1 [27,28]. More importantly, this heterochromatic environment seems to be required for proper expression of clusters and their processing into piRNAs. H3K9me3 is deposited on clusters by the histone methyltransferase SetDB1 [28]. Mutation or knockdown of SetDB1 leads to loss of piRNAs and concomitant upregulation of TEs and sterility. Despite the necessity of a heterochromatic environment for piRNA production, deposition of H3K9me3 and HP1 cannot be sufficient for identification of clusters, as these features are present on many genomic regions that do not yield piRNAs. Interestingly, a germline-specific homolog of HP1, Rhino, also associates with double-stranded piRNA-producing loci and seems to be absent from other genomic loci [29]. Rhino deficiency leads to loss of germline-specific piRNAs and derepression of transposons. In the nucleus, Rhino colocalizes in distinct areas with the nuclear DEAD-box RNA helicase protein UAP56 that binds to piRNA precursors [30]. Nuclear regions enriched in Rhino and UAP56 localize close to perinuclear nuage granules, the centers for piRNA processing. Based on this observation, it was proposed [30] that Rhino specifies precursor transcripts by promoting their association with UAP56, which then shuttles piRNA precursors to nuage granules (Figure 2a).

Although it seems clear that chromatin has an important role in regulating the expression of piRNA clusters, currently it is not clear whether the chromatin state simply provides a permissive environment for piRNA cluster activity, or whether, at least in some cases, it is by itself sufficient to specify such regions.

### The role of *trans*-generationally inherited piRNAs in defining piRNA-producing loci

Instead of intrinsic features such as specific sequence signals or a unique chromatin signature, the loci that generate germline piRNAs in *Drosophila* might be defined by a molecular memory provided by the pool of piRNAs inherited from the previous generation. Piwi proteins and the associated piRNAs expressed in the maternal germline during oogenesis are deposited into the developing egg and are present in the early embryo before the start of zygotic transcription [31]. In *Drosophila*, such maternally inherited piRNAs are essential for effective piRNA-mediated silencing and fertility of the progeny. Indeed, recent studies of a long-known phenomenon called hybrid dysgenesis showed that the absence of piRNAs inherited from the previous generation causes a failure to produce cognate piRNAs in the progeny [32].

The role of *trans*-generationally inherited piRNAs in specifying the activity of piRNA loci in the next generation has recently been revealed by studies of transgenes that generate piRNAs. It was shown that a transgene that generates piRNAs is able to induce piRNA production from another locus that was originally incompetent for piRNA generation [33]. After initial activation, the ability of the recipient locus to produce piRNAs is independent of the inducer locus and stable over multiple generations, but only if the activated recipient locus is inherited from the maternal side. The ability to induce piRNA generation requires sequence similarity between the inducer and recipient loci and is an example of a phenomenon called paramutation, the ability of one (inducer or paramutagenic) allele to stably change the expression state of (paramutate) the recipient allele. Importantly, the study [33] revealed that maternally deposited piRNAs generated from the paramutagenic allele are sufficient to induce paramutation of the recipient locus by themselves, without the presence of the paramutagenic locus, indicating that the inherited piRNAs provide a molecular signal that drives the paramutation. Although piRNA-induced paramutation has so far been demonstrated exclusively for transgenic loci, it is plausible that native piRNA clusters are similarly paramutated by maternally deposited piRNAs to become active. Overall, paramutation induced by the inherited piRNAs provides an elegant explanation for the problem of selecting genomic regions for piRNA generation. Furthermore, this mechanism guarantees that the pool of piRNAs produced in each generation is adequate to repress transposons (at least the maternally inherited ones), as only flies that are successful at TE repression are fertile and able to transmit their piRNAs to the progeny.

Two possible mechanisms can be envisioned for how piRNAs inherited from the mother might act to start generation of new piRNAs in the progeny (Figure 2b). They might initiate the Ping-Pong amplification loop and thereby lead to processing of cluster transcripts expressed in the embryo that otherwise would be left unprocessed. Alternatively, inherited piRNAs might induce the establishment of the distinct chromatin state required for transcription and processing of piRNA precursors on target genomic loci. Indeed, recent studies demonstrated that the nuclear Piwi protein induces deposition of the H3K9me3 mark on target loci, probably through recognition of nascent transcripts by Piwi-bound piRNAs followed by recruitment of the chromatin-modifying machinery [4,5,34]. The two mechanisms are not mutually exclusive, and *trans*-generationally inherited piRNAs can activate piRNA generation both by inducing chromatin changes and by initiating precursor processing through the Ping-Pong cycle. It should be noted that these mechanisms can only regulate piRNA production in germ cells but not in follicular cells of the *Drosophila* ovary: piRNAs specific to follicular cells are not deposited into the embryo and thus cannot serve as a template for either mechanism. In addition, follicular cells also lack components necessary for the Ping-Pong amplification loop.

Taken together, recent studies implicate chromatin state and *trans*-generationally inherited piRNAs as two components that are required for activity of regions that generate piRNAs. Chromatin and inherited piRNAs can act in concert to identify genomic loci for piRNA generation, with chromatin state licensing certain genomic regions and inherited piRNAs providing further direction using both the Ping-Pong cycle to initiate processing and a feedback loop to ensure that the compatible chromatin state is preserved.
